# Chronic Mild Stress Induces Fluoxetine-Reversible Decreases in Hippocampal and Cerebrospinal Fluid Levels of the Neurotrophic Factor S100B and Its Specific Receptor

**DOI:** 10.3390/ijms11125310

**Published:** 2010-12-21

**Authors:** Han Rong, Gaohua Wang, Tiebang Liu, Huiling Wang, Qirong Wan, Senghong Weng

**Affiliations:** 1 Department of Psychiatry, Renmin Hospital, Wuhan University, Jiefang Road 238#, Wuhan 430060, China; E-Mails: ronghansz@126.com (H.R.); rotsss@126.com (H.W.); wqrooo@yahoo.cn (Q.W.); wshjjkk@126.com (S.W.); 2 Department of Biological Psychiatry, Shenzhen Institute of Mental Health, Shenzhen Kangning Hospital, Cuizhu Road 1080#, Shenzhen, 518020, China; E-Mail: liutbsz@yahoo.cn

**Keywords:** chronic mild stress, S100B, RAGE

## Abstract

Chronic mild stress (CMS) affects the hippocampal structure and function in the rat. S100B, a calcium-binding protein secreted by astrocytes, has been shown to be increased in serum of patients with depression and associated with good therapeutic response and clinical outcome. This work aimed to study the impact of CMS and fluoxetine on depressive-like behaviors in rats, as well as the concomitant expression of the astroglial protein S100B and of its receptor RAGE (receptor for advanced glycation end products) in the hippocampus and Cerebrospinal fluid of the same group of animals. S100B and sRAGE (circulating soluble form of RAGE) were measured in CSF by ELISA, and S100B and RAGE were measured in hippocampal slices by Western blot. Our study has demonstrated that stress and depression decrease S100B and RAGE/SRAGE expression and antidepressant treatment reverses or blocks these effects. This result suggested that S100B/RAGE interactions may be involved in the development and maintenance of depression and may play an important role in the mechanism of antidepressants’ therapeutic action.

## 1. Introduction

Recently, the role of glia cells in the pathobiology of depression is becoming apparent, highlighted recently by the postmortem finding of a reduced number of glial cell in patients with major depressive disorders [1]. The gila-derived neurotrophic marker S100B, a calcium-binding protein produced mainly by astrocytes, has been shown to be increased in the serum of patients with a melanchlolic subtype of major depression. It has been demonstrated that increased S100B serum concentrations in major depression were associated with good therapeutic response and clinical outcome [2,3], which suggests a role of S100B in the modulation of the course of depression. There is some evidence that rats exposed to chronic restraint stress have a decrease of neurogenesis in the dentate gyrus of hippocampus, which is accompanied by a non-significant increase in the percentage of cells with astrocytic phenotype identified by S100B immunostaining [4]. Previous reports showing that fluoxetine treatment increases the content of S100B in the hippocampus of aged mice [5], and that hippocampal astrocyte cultures and hippocampal slices exposed to fluoxetine increase their S100B secretion by a mechanism independent of serotonin [6]. Recently, the receptor for advanced glycation end products (RAGE) has been suggested to be the putative receptor for S100B [7]. RAGE plays a crucial role in several disease processes, such as diabetes, inflammation, and neurodegeneration [8,9], Multiple roles of RAGE in neuronal differentiation and neurite outgrowth have been reported [9].

S100B is unique in that it can engage RAGE in neurons at low and high concentrations with trophic and toxic effects. S100B/RAGE interactions might have important consequences during development and in tissue homeostasis as well as in inflammatory, degenerative and tumor processes [7,8]. Soluble RAGE (sRAGE), lacking the transmembrane and signaling domains, is generated by alternative splicing (endogenous secretory RAGE/esRAGE) or by matrix-metalloproteinase induced ectodomain shedding [10].

We purpose that S100B/RAGE (sRAGE) interactions might participate in the pathobiology of depression and could be a target for the action of antidepressant drugs. So, the primary aim of the present study was to investigate the changes in the expression of S100B and RAGE/SRAGE in hippocampus and CSF of rats exposed to chronic mild stress, and the effect of a four week treatment with the antidepressant fluoxetine. We used quantitative Western immunoblotting and ELISA to investigate the content of S100B and RAGE/SRAGE in the hippocampus and CSF of rats. Furthermore, we used open field tests and sucrose preference tests to explore the effects of CMS on activity and anhedonia of rats and correlated these findings with protein changes observed in the hippocampus.

## 2. Results and Discussion

### 2.1. Effects on Sucrose Consumption, Body Weight and Open-Field Test

**Figure 1.**
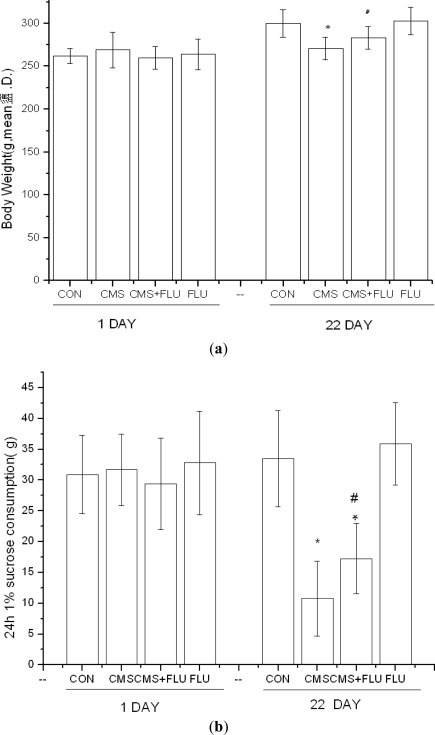

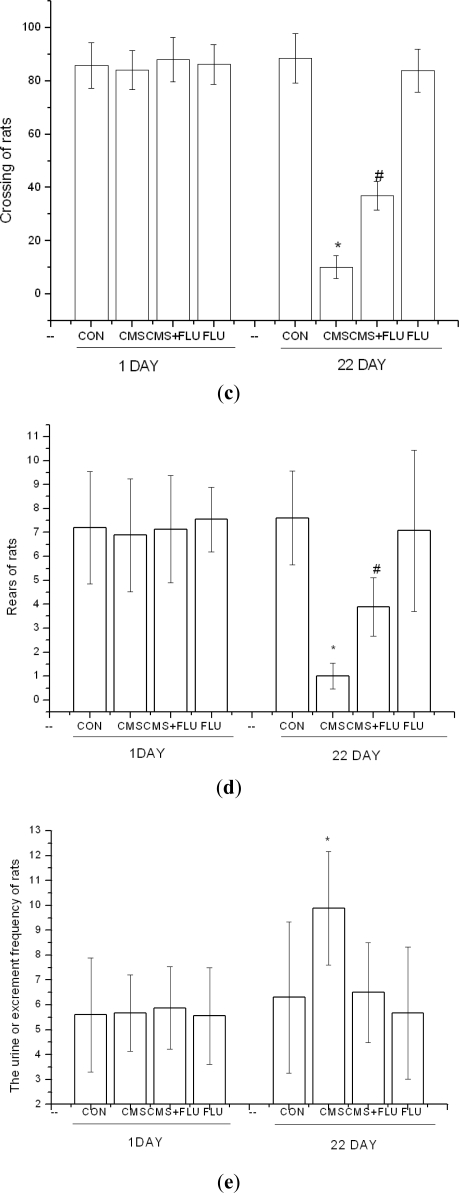
Effects on sucrose consumption, body weight and open field test. * *p* < 0.001 *vs.* control, ^#^ *p* < 0.01 *vs.* CMS (one-way ANOVA followed by Bonferroni test). Results are expressed as mean ± S.D. (**a**) 24 h 1% sucrose consumption of rats before and after CMS; (**b**) Body weight of rats before and after CMS; (**c**) Crossing of rats before and after CMS; (**d**) Rears of rats before and after CMS; (**e**) The urine or excrement frequency of rats before and after CMS.

Sucrose consumption and body weight Figure 1 (a) and (b), crossing (c), number of rears (d), and the urine or excrement frequency (e), were measured twice during the experimental period. As shown, no differences were found among four groups of rats before CMS. The three weeks of chronic stress induced a marked decrease in the sucrose consumption, body weight, crossings and rears of open field activity, but an increase in the urine or excrement frequency in the stressed rats compared to the non-stressed group. [*F* (3,36) = 7.605, *p* < 0.001; *F* (3,36) = 4.132, *p* < 0.01; *F* (3,36) = 80.42, *p* < 0.001; *F* (3,39) = 98.502, *p* < 0.001; *F* (3,36) = 69.369, *p* < 0.001].

### 2.2. Effects of CMS and Fluoxetine Treatment on Expression of S100B and sRAGE in the CSF

**Figure 2.**
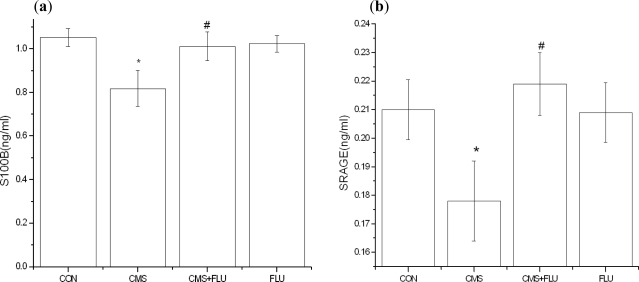
Effects of CMS, CMS + Flu and Flu on the levels of S100B and sRAGE in the CSF of rats. (* *p* < 0.05 *vs.* control, *^#^* *p* < 0.05 *vs.* CMS). Results are expressed as mean ± S.D. (**a**) Effects of CMS, CMS + Flu and Flu on the Levels of S100B in the CSF of rats; (**b**) Effects of CMS, CMS + Flu and Flu on the levels of SRAGE in the CSF of rats.

CSF S100B and sRAGE expression in control rats (Con) and rats subjected to chronic mild stress (CMS), chronic mild stress + fluoxetine (CMS + Flu) or fluoxetine (Flu) (Figure 2a and 2b). CMS significantly downregulated S100B and sRAGE levels, an effect prevented by the concomitant administration of Fluoxetine (CMS + Flu). Figure 2a and 2b [S100B one-way ANOVA, *F* (3,36) = 5.471, *p* = 0.003, SRAGE one-way ANOVA, *F* (3,36) = 6.033, *p* < 0.02, followed by the Bonferroni test, *p* < 0.05].

### 2.3. Effects of CMS and Fluoxetine Treatment on Expression of S100B and RAGE in the Hippocampus

**Figure 3.**
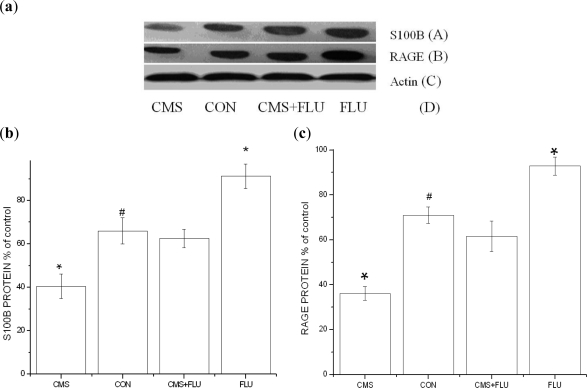
Representative Western blot bands and densitometric analyses of the bands of S100B and RAGE in the hippocampus. Results are expressed as mean ± S.D. (**a**) Representative Western blot bands and densitometric analyses of the bands for: (A) S100B, (B) RAGE, (C) Actin and (D) CMS, Control, CMS + FLU, FLU; (**b**) Effect of CMS and FLU on hippocampus S100B levels (* *p* < 0.05 *vs.* control, *^#^* *p* < 0.05 *vs.* CMS, followed by the Bonferroni test); (**c**) Effect of CMS and FLU on hippocampus RAGE levels (* *p* < 0.05 *vs.* control, *# p* < 0.05 *vs.* CMS, followed by the Bonferroni test).

Photomicrograph illustrates an example of S100B and RAGE protein analysis using Western blot (Figure 3a). Graphic representation of S100B and RAGE protein expression in control rats (Con) and rats subjected to chronic mild stress (CMS), chronic mild stress + fluoxetine (CMS + Flu) or fluoxetine (Flu). CMS significantly downregulated S100B and RAGE levels (*p* < 0.05), an effect prevented by the concomitant administration of Fluoxetine (CMS + Flu) (*p* < 0.05). Administration of Flu alone (under stress-free conditions) shows a significant increase in S100B and RAGE levels (*p* < 0.05) (Figure 3b and 3c).

### 2.4. Disscusions

We used CMS as it has been shown to produce behavioral changes that are similar to human depression and considered to be a valid and useful experimental model of depression [11,12]. Concurrently, the rats were treated with fluoxetine which was reported to have the ability to reverse stress-induced changes. Our present study reveals that chronic stress and fluoxetine treatment exert opposing effects on the regulation S100B expression in CSF and hippocampus of rats. S100B levels are decreased in the CSF and hippocampus of rats after chronic unpredictable stress when compared with normal controls. The findings may be paralleled with the recent work by Sakatani *et al.* who reported S100B is released in a neural activity dependent manner, possibly through activation of mGluR3 and some neural activity are influenced in S100B knockout mice [13,14]. This result is also consistent with previous studies which show that S100B expression in serum was increased rather than decreased after CMS and chronic antidepressant treatment reversed CMS induced increased S100B expression in serum [2,3]. This report indicates that central and peripheral S100B changes do not occur in parallel and that plasma S100B levels can’t be a surrogate marker for CNS and hippocampal S100B levels.

In fact, it is difficult to reconcile the reduction of plasmatic S100B caused by antidepressants in humans with the observation that fluoxetine treatment enhances the content of S100B in the hippocampus of aged mice [5], and that it promotes the secretion of S100B by hippocampal astrocyte cultures and hippocampal slices through a mechanism independent of serotonin [15]. A novel theory of depression has been formulated that proposes a deficiency in adult brain neurogenesis as the pathobiological basis of depression [16–18]. Correlated with this, there is extensive evidence that demonstrates an involvement of S100B in neurogenesis. In serotonergic neurons, S100B acts as a trophic factor and a neurite outgrowth and differentiation promoter and even appears to neutralize neurotoxic challenges [18]. Most importantly, it stimulates the outgrowth of neuritis and promotes the survival of neurons during development. S100B has been shown to be released by astrocytes to the extracellular space and to affect astrocytes in an autocrine manner and neurons in a paracrine manner [19–21]. Once released, effects of S100B on target cells depend on its concentration. At nanomolar concentrations, S100B is trophic in that the protein stimulates neurite outgrowth [14], enhances survival of neurons during development, and, after injury, prevents motor neuron degeneration in newborn rats after sciatic nerve section [12,18].

Our present study also reveals that chronic stress and fluoxetine treatment exert opposing effects on the regulation of RAGE expression in the hippocampus of rats. RAGE levels are decreased in the hippocampus of rats after chronic unpredictable stress when compared with normal controls.

It has been suggested that S100B interacts with the RAGE on the cell surface and RAGE has been proposed as a signal transducing receptor for both trophic and toxic effects of S100B [8,9]. Treatment with S100B significantly elevated RAGE-mRNA levels in the rat hippocampal neurons cultures, lending support to the notion that S100B acts as a direct RAGE ligand in primary neurons [9]. At the low levels normally found in the brain extracellular space, S100B acts as a neurotrophic factor protecting neurons against noxious stimuli and, also, binding of S100B to RAGE in neurons has been shown to result in the up-regulation of the antiapoptotic factor, Bcl-2, in a Ras-MEK-ERK1/2-NF-kB dependent manner [7], which strongly supports the notion that S100B might protect neurons against noxious stimuli and promote neuronal repair during the very early phase following brain insults and might contribute to neuronal differentiation. Ultimately, such actions of S100B and RAGE could increase adult neurogenesis. RAGE may also participate in neuronal differentiation and neurite outgrowth. RAGE has been previously shown to mediate neurite outgrowth on amphoterin coated matrices [7]. Furthermore, studies have shown that RAGE mediated neurite outgrowth can be abolished by deleting the cytoplasmic domain of RAGE [7,9]. Taken together, our findings support the possibility that decreased expression of S100B and RAGE in hippocampus contributes to the deficiency in neurogenesis that underlies chronic mild stress.

Malberg *et al.* show that chronic fluoxetine could be effective in increasing S100B levels in rat hippocampus [22]. Our result is in keeping with a previous study; moreover, Malberg *et al*. treated unstressed rats with fluoxetine while we treated stressed rats with fluoxetine. It has been reported that chronic antidepressant treatment increases cell proliferation and granule cell survival and is able to reverse the stress-induced decrease of hippocampal neurogenesis [11,23]. Thus, the observed S100B and RAGE changes following 21 days fluoxetine treatment, strongly suggest that chronic fluoxetine could be effective in recovering the decrease of S100B and RAGE levels induced by stressors in rat hippocampus, and chronic fluoxetine administration has the potential to affect neuronal and synaptic remodeling via changes in S100B/RAGE dynamics.

In this study a decrease, rather than an increase, in CSF s100B and sRAGE levels was observed after CMS in the rats. The stressed animals that were chronically treated with fluoxetine showed increased expression of CSF s100B and sRAGE, Soluble RAGE (sRAGE), lacking the transmembrane and signaling domains, is generated by alternative splicing (endogenous secretory RAGE/sRAGE) or by matrix-metalloproteinase induced ectodomain shedding [10]. In human subjects, studies suggest that the absolute levels of soluble RAGE may correlate with chronic disease states and their severity, and that soluble RAGE levels may be mutable consequent to therapeutic interventions [10]. A number of studies have suggested that administration of soluble RAGE is protective against oxidative stress and neuronal death [10,24], and low levels of sRAGE have been indicated as a possible new marker of low-grade inflammation [25]. Given the beneficial role of sRAGE in metabolic and inflammatory diseases, we hypothesized a similar effect in depression, possibly related to a self-limiting regulatory mechanism contributing to a normalization of S100B levels. Thus, it is plausible that soluble RAGE may represent a therapy for disease states in which the ligands of RAGE accumulate. Many questions remain to be addressed, such as whether soluble RAGE display identical affinity for RAGE ligands and whether sRAGE levels in CSF or plasma could benefit or biomark depression. No doubt, as the literature is abounding with studies examining soluble RAGE in the human subject in homeostasis and disease, the answers to these questions are soon to be revealed.

Although stress resilient animals are often found [26,27], we did not find any of the stressed resilient animals in this study. As in previous studies [28,29], this study confirmed that CMS caused reduction in sucrose consumption, motor and exploratory activities. Furthermore, the stressed animals treated with fluoxetine showed a significant increase in sucrose consumption, motor and exploratory activities. These behavioral changes were accompanied with decreased S100B and RAGE/sRAGE levels, and increased S100B and RAGE/sRAGE levels, respectively. These findings could be indicative of hippocampal and CSF S100B and RAGE/sRAGE changes which might in turn contribute to the behavioral changes. However, although these findings showed a correlation between S100B/RAGE interactions and behavioral changes in rats, it should be underlined that we cannot conclude whether S100B/RAGE interactions play a causal or only a correlative role with the observed changes. A behavioral study found some behavioral phenotypes with RAGE KO mice, and this study suggested deletion of RAGE causes hyperactivity and increased sensitivity to auditory stimuli in mice [30]. The relationship between changes in behavioral phenotype and S100B/RAGE interactions needs to be further investigated.

The results of this study can be interpreted as follows: It can be assumed that a decrease in levels of S100B and RAGE/sRAGE in hippocampus and CSF of chronic stress rats indicates a neuroprotective activity that is evidence that certain mechanisms of neuronal plasticity and neurogenesis may be functionally impaired in major depression. Treatment with Fluoxetine can prevent the impaired process of neuronal plasticity and neurogenesis through actions of S100B and RAGE. S100B/RAGE interactions involved in development and maintenance of depression and may play an important role in the mechanism of the antidepressants therapeutic action.

## 3. Experimental Section

### 3.1. Animals

Forty adult male Sprague-Dawley rats from the Wuhan University’s Animal Center (5–7 weeks old, weighing 250–300 g) were housed with *ad lib* access to food and water, and maintained on a 12-h light/dark cycle (lights on at 7:00 a.m.), at 22 °C with low humidity. All animal experiments were carried out in accordance with the National Institute of Health Guide for the Care and Use of Laboratory Animals (NIH publication 8023, revised 1996) and with the approval of the local Animal Use Committee.

After one week of habituation, rats were randomly divided into four groups of 10 animals each. The group I (Con) received saline (1 mL/kg), group II (FLU) received Fluoxetine (10 mg/kg) + saline (1 mL/kg), group III (CMS) received CMS + saline (1 mL/kg) and group IV (CMS + FLU) received CMS + Fluoxetine (10 mg/kg) + saline (1 mL/kg). Fluoxetine (10 mg/kg) (Sigma, St. Louis, MO, U.S.) were intraperitoneally administered with a volume of 10 mL/kg repeatedly. The repeated treatment was performed once a day. For the CMS + FLU and FLU rats, the administration of antidepressants was from day 1 to day 21. Doses of 10 mg/kg of fluoxetine were chosen because, at such a level, fluoxetine has been reported to show antidepressant action in previous work [28,31]. The weight of rats and consumption of saccharose was monitored during the periods of stress as an index. Open field test was used to observe the behavior of rats on the day before stimulation and on the 22nd day during experiment.

### 3.2. The CMS Model and Behavior Test

The CMS model of depression is accepted as a valuable method for evaluating antidepressant effects in animals. In this model, animals subject to various stressors such as unpredictable order and stimulating conditions in the natural environment, show many behavioral, biochemical and physiological impairments, which parallel the symptoms of depression in human. In the present study, the CMS procedure consisted of a variety of unpredictable mild stressors including repeated periods of 45°cage tilt; two 2-h periods of separated housing; one overnight period of limited access to food (12-h separation of rats from their chow by direct touching of rat by glass clapboard with holes of 3 mm diameter, yet without any reduction in the actual daily food ration); one period of continuous overnight illumination; and one overnight period in a soiled cage (50 mL of water/L of sawdust bedding). Animals were also placed on a reversed light/dark cycle from Friday evening to Monday morning. These stressors were scheduled over a one-week period and repeated throughout the 3-week experiment [31,32]. In contrast to previous procedures in rats, nociceptive stressors were excluded, and only environment and social disturbances were applied. The non-stressed control animals were housed in normal conditions without any influences. The injections of fluoxetine or saline were applied just after the stressing procedures.

All rats were tested for sucrose consumption and given 1% (w/v) sucrose solution in water for 24 h in place of their regular drinking water in their home cages, following 24 h period of water deprivation on the 1st and the 22nd day, respectively. The bottles were weighed prior to being given to the rats and at the conclusion of the test (7:00 a.m. the next morning). On the 1st and 22nd day, the weight of rats was recorded and rats were submitted to the open-field test. The open-field was a 50 × 25 × 50 chamber made of brown polywood with frontal glass wall. The floor of the open-field was divided into 12 equal squares by black lines. Animals were placed on the left rear quadrant and left to explore the arena. Animals were observed for 3 min to record the number of rears (animal on hind limbs), crossing (grid boxes entered) and the urine or excrement frequency.

### 3.3. Tissue Isolation, Western Blot and ELISA

After the behavior experiment, CSF samples (used for ELISA determinations) were obtained by magna cistern puncture and hippocampus (used for quantitative Western immunoblotting determinations) were isolated through the stereo toxic atlas of the rat brain and then were snap-frozen. For determination of S100B and RAGE protein level, protein extracts were obtained from the hippocampus of brain according to the following protocol. Each assay sample consisted of the hippocampus from one rat. Each sample was weighed and homogenized in 1.5 mL of sample buffer (0.01 M Tris-HCl buffer (pH 7.6) containing 0.25 M sucrose, 0.1 M NaCl, 1 mM EDTA, and 1 mM phenylmethylsulfonylfluoride) at 4 °C. Supernatant, after 12,000 rpm centrifugation for 10 min, was used for Western blotting. Protein concentration was determined according to the method of Bradford. Samples were dissolved with equal volume of loading buffer (0.1 M Tris-HCl buffer (pH 6.8) containing 0.2 M DTT, 4% SDS, 20% glycerol and 0.1% bromophenol blue), separated on 10% SDS-PAGE and then electrotransferred at 100 V to Immun-Blot PVDF membrane for 1 h at 4 °C. Membranes were blocked in Tris-Buffered Saline Tween-20 (TBST) containing 5% non-fat dried milk overnight at 4 °C before incubation for 2 h at room temperature. Blots were then incubated with either one of the following: Rabbit anti-S100B polyclonal antibody (1:1000, Santa Cruz), or rabbit anti-RAGE polyclonal antibody (1:1000, Santa Cruz) for 1.5 h at room temperature. The blot was washed several times with TBST and then incubated with the secondary antibody, peroxidase-conjugated anti-rabbit IgG dilution (1:10,000, Santa Cruz). Signal detection was performed with an enhanced chemiluminescence kit. The relative changes of the intensity of each immunoreactive band were captured and analyzed using GeneSnap Image Analysis Software (Syngene, U.K.). CSF S100B was measured by immunoluminometry (E0567rS100B ELISA kit, ELAabscience, Wuhan, China) according to the manufacturer’s instructions. CSF sRAGE was determined by sandwich enzyme-linked immunosorbentassay (SK0011203sRAGE ELISA kit, Yunhan Biology, Shanghai, China) according to the manufacturer’s instructions.

### 3.4. Statistical Analysis

Results are expressed as mean ± S.D and analyzed by SPSS 11.0. Repeated measures analysis of variance (ANOVA) was used for *post-hoc* analysis for differences between groups. *p* < 0.05 was considered statistically significant.

## 4. Conclusions

This study is the first to demonstrate that S100B/RAGE interactions may be involved in the development and maintenance of depression and that it may play an important role in the mechanism of antidepressants’ therapeutic action.
